# The effect of fertilizing soils degraded by the metallurgical industry on the content of elements in *Lactuca sativa* L.

**DOI:** 10.1038/s41598-021-83600-7

**Published:** 2021-02-18

**Authors:** Alicja Kicińska, Justyna Wikar

**Affiliations:** Department of Environmental Protection, Faculty of Geology, Geophysics and Environmental Protection, AGH University of Science and Technology, Mickiewicza 30 av., 30-059 Kraków, Poland

**Keywords:** Environmental monitoring, Plant sciences

## Abstract

The aim of the study was to determine the content of macroelements (Ca, K, P, S, Mg) and microelements (Fe, Cr, Ni, Mn, Zn, Pb, Zn, Ti) in the leaves of *Lactuca sativa* grown in soils contaminated by the mining and metallurgical industry. The plants were cultivated using four fertilization variants: (*a*) unfertilized soil, (*b*) mix of straight fertilizers, (*c*) multinutrient fertilizer and (*d*) organic fertilizer, namely granular cattle manure. The study also involved an analysis of metal accumulation degree in the edible parts of lettuce by means of calculating a bioaccumulation index—transfer factor (*TF*). The analysis of the impact of fertilization on the content of the elements in the edible parts of fertilized versus unfertilized lettuce demonstrated that phytoavailability of the metals was most effectively limited by the multinutrient fertilizer and the mix of straight fertilizers. The organic fertilizer proved to be the least effective. The highest *TF* values (> 0.1) were recorded for macroelements, which denotes their intense and moderate accumulation. Poor bioaccumulation was observed for Cr, Mn, Ni and Zn (0.01 ≤ *TF* < 0.1), whereas in the case of Fe, Pb and Ti—trace bioaccumulation or no bioaccumulation was found (*TF* ≤ 0.01).

## Introduction

Due to changes in people’s eating habits over the last 10 years, it is now recommended to include increasingly more vegetables and fruits in one’s daily diet^1^. Plants are indeed an important source of vitamins, minerals and fibre. However, they can also be a significant source of toxic substances, i.e. nitrates, heavy metals and metalloids, which can be accumulated in their edible parts in amounts that pose a threat to human health and even life^2–7^. Therefore, the knowledge of plant cultivation and fertilization methods as well as quality control play an important role in the agricultural production of vegetables and fruit^8,9^.

People growing vegetables in small vegetable gardens or allotments are particularly exposed to the consumption of produce inferior in quality. The lack of awareness of the pollutant load found in agricultural soil fosters an erroneous belief about the superiority of homegrown produce over that bought at markets or obtained from the so-called mass production. Crops fertilized in an improper manner with mineral fertilizers are also dangerous for health, mainly when excessive amounts of fertilizers are used^10–12^.

For their proper development, plants need bioelements occurring in significant quantities (so-called macroelements) and in trace amounts (so-called microelements). Both groups, absorbed in appropriate amounts, constitute indispensable protein-forming components, building plant cell walls or chlorophyll. Also, they are part of the compounds responsible for the proper course of photosynthesis and osmoregulation. The first group of these components includes: N, P, S, K, Mg and Ca, whereas the other one comprises: Fe, Zn, Mo, Cu, Mn, Cr, Ni and Bo. The activity of bioelements is mainly associated with the regulation of biochemical processes at the cell level and the activation of plant enzymes. Bioelements also include metals, which in excessive amounts can be harmful or even toxic to plants^13–15^. The absorption of metals by plants is a varied process, which to a large extent depends on the species affiliation, age of the organism, its condition and environmental factors such as climate, terrain, sunlight, soil pH or symbiosis with soil microorganisms^16,17^. In the case of crops, leafy vegetables such as lettuce, spinach or parsley have the highest potential to accumulate excessive amounts of metals. They can absorb heavy metals not only from the soil, but also through the foliar route, through atmospheric dust fall containing a high pollutant load^18–20^. Root and brassica vegetables are less sensitive in this respect. The degree of metal accumulation is varied and largely depends on the plant organ. The metal content in individual organs generally increases in the following order: stems < leaves < fruit < seeds^21^. When planning the cultivation of particular types of vegetables, attention should be paid not only to the degree of soil contamination but also to the presence of minerals, especially those fertilizer-derived^9,22,23^, as they are relatively easily absorbed by the root system. Being chiefly unselective, it is the cell wall of the roots that is responsible for the uptake and accumulation of metal ions^24,25^. Along with the increase in the concentration of metals in the soil, their content in plant organs generally increases, which is due to the lack of barrier formation hindering the selective passage of ions^10,26^. The highest phytoavailability is observed in the easily soluble forms of metals. They occupy exchangeable positions or are bound, mainly with carbonates, by weak ionic forces^27,28^. An additional factor affecting the accumulation of metals in plants may be the deficiency of certain microelements in the soil, which may contribute to more intense absorption of other elements, including heavy metals, by plants^21,24^. The transfer of metals from the soil to the plant is considered to decrease as metal bioavailability decreases^29–31^.

Bearing these facts in mind, soil samples were taken in areas affected by centuries of metallurgical industry related to the extraction and processing of Zn–Pb ores as well as Fe ores, and then subjected to the following analyses:

- the total content of macro—(Ca, K, P, S, Mg) and microelements (Fe, Cr, Ni, Mn, Zn, Pb, Zn, Ti) in the leaves of lettuce grown in these soils was determined using various types of fertilization (mix of straight fertilizers, multinutrient fertilizer, organic fertilizer) and no fertilization;
- the influence of various types of fertilization on the content of selected elements in edible parts of lettuce in relation to plants grown in unfertilized soils was examined;
- the bioaccumulation index—transfer factor (*TF*) values were calculated for the lettuce;
- the obtained content of metals was compared to the permissible levels defined by legal regulations and those provided in the literature data.

## Study area and material

**Figure 1:**
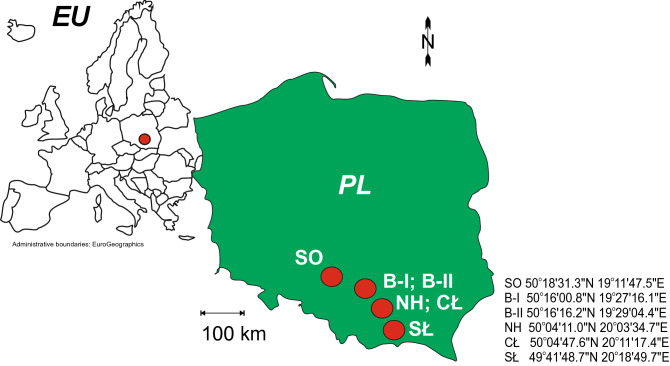
Sampling sites. The map was created by the authors using CorelDRAW2020 software, ver. 22.1.1.523; based on: https://ec.europa.eu/eurostat/documents/ © EuroGeographics for the administrative boundaries.

Soil samples, 25 kg each, were taken from 5 locations in southern Poland (CEE) to conduct pot experiments. Samples with symbols B-I and B-II (Fig. 1) were collected in Bukowno, from areas subject to centuries of mining and processing of Zn–Pb ores. The sample marked with the SO symbol (Fig. 1) was taken from Sosnowiec, a place associated with the exploitation of hard coal and manufacture of steel products. Samples NH and CŁ were taken in Nowa Huta and Cło, from sites associated with Fe ore processing and manufacture of steel products. In addition, a control sample was taken in Słopnice (SŁ symbol). The area of Słopnice is characterized by the lack of influence of any industry, and most of the land is used for agriculture.

Soil samples from each location were taken from five independent sites, 10 m apart, from 50 cm × 50 cm pits at a depth of 0–25 cm below the ground level, weighing approx. 5 kg each. The material was placed in ventilated PVC containers, and then transported to a greenhouse with constant temperature (approx. 21 °C) and humidity (soil moisture index ranged from 60 to 80%). The pot experiment involved cultivation of lettuce (*Lactuca sativa* L*.*) in 6 soil samples: B-I, B-II, SO, NH, CŁ and SŁ, with the use of four fertilization variants: (*a*) unfertilized soil, (*b*) mix of straight fertilizers, (*c*) multinutrient fertilizer and (*d*) organic fertilizer, which was granular cattle manure. In each fertilization variant, 30 lettuce seedlings were sown. The seedlings were planted in pots with the volume of 5 dm^3^ and a side of 50 cm. All types of fertilizers were applied for 10 days before planting the seedlings.

A mix of straight fertilizers (variant *b*) was used in the following doses (per pot): 0.6 g nitro-chalk with magnesium (27% N), 0.4 g enriched superphosphate (40% P_2_O_5_) and 0.5 g potassium sulphate (50% K_2_O), in order to obtain about 80 kg N/ha, 72 kg P_2_O_5_/ha and 120 kg K_2_O/ha, respectively. The “Azofoska” multinutrient fertilizer and granular cattle manure (variants *c* and *d*) were respectively dosed as 1 g and 15 g per pot, in accordance with the dosage recommended by the manufacturer.

The cultivar chosen for the experiment was Sprinter 5G Standard early butterhead lettuce suitable for cultivation in plastic tunnels, under Agryl covers and without any covers. This cultivar forms light green heads which are well closed from below. The plants were watered with tap water throughout the entire growth period, as needed. They were provided with appropriate light exposure for minimum 12 h a day and grown in a greenhouse for 2 months (July–August 2019).

## Study methods

After the two-month growth period, lettuce leaves were harvested, thoroughly washed 3 times with distilled water, and then dried. The material was ground in an electric mill and dried at 105 °C for 1 h to obtain constant mass. Extraction was carried out in a mixture of hydrogen peroxide (30% H_2_O_2_) and concentrated nitric acid (65% HNO_3_), in a 1:10 ratio of solid phase to solution^31^. The decomposition took place in SCP Science microwave oven type DigiPREP HT at 105 °C for 2 h. Extraction solutions were passed through filters and their volume was adjusted to 50 cm^3^ with distilled water. The concentrations of the following elements: Ca, Cr, Fe, K, Mg, Mn, Ni, P, Pb, S, Ti and Zn in the extracts obtained were determined using ICP-MS (Elan model), at a certified geochemical laboratory (certificate no. AB1050) of the AGH University of Science and Technology in Krakow. The Certipur Certified Reference Material (CRM048, Lot LRAB1604) was used as a standard. The precision of element determination was 8%, while the accuracy (AO) ranged between 95.2 and 98.2%.

The bioaccumulation index—transfer factor (*TF*) was determined in accordance with formula no. 1. The assessment of the accumulation degree in green plants was carried out in accordance with the recommendation by Kabata-Pendias and Pendias^32^, assuming the following ranges for *TF* values: *TF* < 0.01—no accumulation, 0.01 ≤ TF0.1—poor accumulation, 0.1 ≤ *TF 1.0*—moderate accumulation and 1.0 ≤ *TF*—intense accumulation. The *TF* values were calculated only for unfertilized soils.

1 $$ TF = \frac{{C_{plant} }}{{C_{soil} }}, $$

where C_*plant*_—total concentration of a metal in the edible part of the plant, in dry matter [mg kg^−1^]. C_*soil*_—total concentration of metal in the soil, in dry matter [mg kg^−1^].

The content of elements in the dry matter of lettuce leaves was converted to fresh mass using a conversion factor of 0.085^33^.

Statistical analysis of the results (PCA, cluster analysis, coefficient of variation) was carried out in Statistica *ver*. 13.3 software. Correlation analysis was performed at a significance threshold of *p* < 0.05.

## Results

### The content of macro- and microelements in unfertilized lettuce

Concentrations of macro- and microelements in the fresh mass of lettuce grown in unfertilized soils (variant *a*) vary widely (Table 1). For the sake of proper analysis, Table 1 also contains the content of elements in unfertilized soils. The correlations related to their occurrence and the assessment of the degree of pollution have been described in detail in the paper by Kicińska and Wikar^34^. The focus of the present paper is on the impact of various types of fertilization on the chemical composition of lettuce.

**Table 1:** The total content of elements in (*a*) unfertilized soil (s) and lettuce leaves (l).

Sampling site	Ca	K	Mg	P	S	Cr	Fe	Mn	Ni	Pb	Ti	Zn
	[mg kg^−1^ for soil in dry mass, for lettuce in fresh mass]											
**Industry areas**												
B-I/*a*	(s) 5030.7 (l) 1680.9	924.1 4051.0	2494.7 514.8	1036.5 805.4	808.4 773.8	16.0 0.6	11,273.9 13.6	555.8 6.5	13.2 0.3	373.6 0.4	10,716.9 0.6	1544.6 15.5
B-II/*a*	7389.0 2240.1	821.8 4172.7	2595.4 444.1	1660.4 794.9	1950.9 841.8	15.6 1.0	10,730.1 16.9	842.4 6.8	14.9 0.5	952.6 0.8	6935.4 1.9	3014.1 37.3
SO/*a*	13,435.4 1594.9	1427.8 4283.9	2961.5 335.6	1653.5 599.5	2369.1 721.8	25.3 0.7	14,702.8 18.4	221.4 3.7	21.4 0.4	232.4 0.8	7930.9 5.7	757.7 15.8
NH/*a*	16,439.5 1811.6	3218.0 3033.3	3432.2 492.3	2648.5 835.9	1230.9 707.3	38.6 0.7	21,431.5 43.7	573.4 5.9	28.0 0.4	56.4 0.3	17,916.5 13.2	348.8 7.6
CŁ/*a*	6029.0 1423.7	3349.0 4758.3	2187.3 392.1	3177.1 1074.6	999.5 713.7	29.1 0.6	15,881.4 14.4	642.8 7.6	23.3 0.3	37.5 0.1	23,756.9 1.2	153.5 6.0
**For all samples (n= 25 for soil; n = 150 for lettuce)**												
Av	9664.7 1750.2	1948.1 4059.8	2734.2 435.8	2035.2 822.1	1471.8 751.7	24.9 0.7	14,803.9 21.4	567.2 6.1	20.2 0.4	330.5 0.5	13,451.3 4.5	1163.7 16.4
SD	4471.8 307.9	1110.2 633.4	427.7 73.2	769.9 169.2	592.6 56.8	8.6 0.2	3851.1 12.6	200.6 1.5	5.5 0.1	334.5 0.3	6427.3 5.2	1041.0 12.5
Min	5030.7 1423.7	821.8 3033.3	2187.3 335.6	1036.5 599.5	808.4 707.3	15.6 0.6	10,730.1 13.6	221.4 3.7	13.2 0.3	37.5 0.1	6935.4 0.6	153.5 6.0
Max	16,439.5 2240.1	3349.0 4758.3	3432.2 514.8	3177.1 1074.6	2369.1 841.8	38.6 1.0	21,431.5 43.7	842.4 7.6	28.0 0.5	952.6 0.9	23,756.9 13.2	3014.1 37.3
*V (s)* *(l)*	0.46	0.57	0.16	0.38	0.40	0.34	0.26	0.35	0.27	**1.01**	0.48	**0.89**
	0.18	0.16	0.17	0.21	0.08	0.29	**1.00**	0.07	0.25	**0.60**	**1.16**	**0.76**
**Control site** (***n***= ***5 for soil; n*** = ***30 for lettuce)***												
SŁ/*a*	(s) 1601.8 (l) 1614.9	2361.6 5610.0	4247.4 437.7	1636.1 839.3	1050.2 857.0	48.6 0.7	16,766.3 26.9	547.7 11.6	50.4 0.4	28.8 0.1	6904.5 4.1	81.0 7.1
Natural content in lettuce	–	–	–	–	–	2.3^1,2,3^	425^2^^,3,4^	0.2^3^ 500^2^^,4^	0.1^3^ 1.5^2^ 10^1^	0.1^1^ 0.2^4^ 0.3^2,3^	–	60^2^ 100^3^^,4^ 10^5^

“*a* “ index—unfertilized; (s)—data for soil samples from Kicińska and Wikar (2020), (l)—data for lettuce; “– “ lack data; V- coefficient of variation, bolded V ≥ 0.6;

^1^Islam et al.^31^.

^2^Tasrina et al.^39^.

^3^Latif et al.^33^.

^4^Ramteke et al.^40^.

^5^Kabata-Pendias and Pendias^32^.

The content of the macroelements analysed in lettuce grown in the soils collected from industrial and unfertilized areas decreases in the following order: K > Ca > P > S > Mg, with their mean content being: 4059.8, 1750.2; 822.1, 751.7 and 435.8, respectively (data in mg kg^−1^ fresh mass, *n* = *150*). The highest concentrations of K and P were found in lettuce grown in the soils from Cło. These were 4758.3 and 1074.6, respectively (in mg kg^−1^). The highest content of Ca, Mg and S, on the other hand, was found in plants grown in the soils from Bukowno: 2240.1, 514.8 and 841.8, respectively (in mg kg^−1^) (Table 1).

Comparing these results to the data obtained for lettuce grown in the control soils, it was found that the experimental plants had: higher Ca content (mean content for the control sample is 1614.9 mg kg^−1^_,_ *n* = *30*), comparable Mg and P content (437.7 and 839.3 mg kg^−1^_,_ respectively) and lower K and S content (5610.0 and 857.0 mg kg^−1^_,_ respectively).

Higher Ca content in the soils from Bukowno and Cło stems from higher (compared to other regions) geochemical background associated with the occurrence of mainly carbonate rocks (limestone and dolomite) in the rock substrate.

In the group of microelements, the content of individual elements in the plant matter was significantly lower compared to the macroelements analysed. They occurred in the following decreasing order: Fe > Zn > Mn > Ti > Cr > Pb > Ni, which corresponds to the following mean values calculated for all industrial areas analysed: 21.4, 16.4, 6.1, 4.5, 0.7, 0.5 and 0.4 (data in mg∙kg^−1^ fresh mass, *n* = *150*) (Table 1).

The highest content of Cr, Ni, Pb and Zn, amounting to: 1.0, 0.5, 0.8 and 37.3, respectively (in mg∙kg^−1^) was found in plants grown in the soils from Bukowno (the area of exploitation and processing of Zn–Pb ores), while lettuce grown in the soils collected in Nowa Huta (the area of Fe ore processing and steel products manufacture) presented the highest content of Fe, Mn and Ti: 43.7, 7.6 and 13.2, respectively (in mg kg^−1^).

Comparing the microelement content in lettuce grown in the industrial soils to the results obtained for lettuce grown in the control soils, it was observed that the mean content was: 2–4 times higher Pb, comparable for Zn, for Cr, Ni, Ti, and almost 2–3 times lower for Mn. In the case of Fe, lower content was found in nearly all sites, except for Nowa Huta (NH, where the Fe content was 43.7 mg kg^−1^). For comparison, the Fe content in lettuce grown in the control soils was two times lower, 21.4 mg kg^−1^, while the mean values for the other industrial areas (Bukowno and Sosnowiec) were 13.6 and 18.4 mg kg^−1^, respectively.

The higher Mn content in the control soils, and thus in lettuce, can be explained by the occurrence of numerous Fe–Mn concretions in the Carpathian Flysch area, which is located in the bedrock of the control site (i.e. in Słopnice). The high content of this element in comparison with other areas does not indicate pollution, but rather an elevated, natural geochemical background reported in other publications^35^.

The Zn content in lettuce grown in the soils from Bukowno was over 4 times (B-I) and over 6 times (B-II) higher than the 44–73 mg kg^−1^ DM range for Poland^32^. Undoubtedly, such high concentrations are associated with the “enrichment” of soil with this element due to the exploitation and processing of Zn–Pb ores taking place in this area. Pb content was also higher. According to the abovementioned authors, the values noted for CEE countries fall within the range of 4–11 mg kg^−1^ DM, while in Bukowno they were over 2 times higher. The Cr content in lettuce grown in the soils from the Bukowno region (B-I and B-II) was nearly 10 times higher than the value provided by these authors (0.8 mg kg^−1^ DM). Mn levels were also higher. On the other hand, the Ni content in the leaves of lettuce grown in the soils from Bukowno fell within the range given by Kabata-Pendias and Pendias^32^, amounting to 1–3.8 mg kg^−1^ DM for Poland.

Comparing the content macro- and microelements with the WHO criteria to be met by food^4,5,36^, it was found that the permissible levels were significantly exceeded for Pb (lettuce grown in the soils from Bukowno, Sosnowiec and Nowa Huta) and Zn (lettuce grown in the soils from Bukowno and Sosnowiec).

In the micronutrient group, a large variation in the occurrence of the elements was observed in individual lettuce samples. This is especially the case for Ti, Fe, Zn and Pb. The calculated coefficient of variation (*V*) for these elements was: 1.16, 1.00, 0.76 and 0.60, respectively (Table 1). This points to unnatural, definitely anthropogenic origin of these elements.

**Figure 2:**
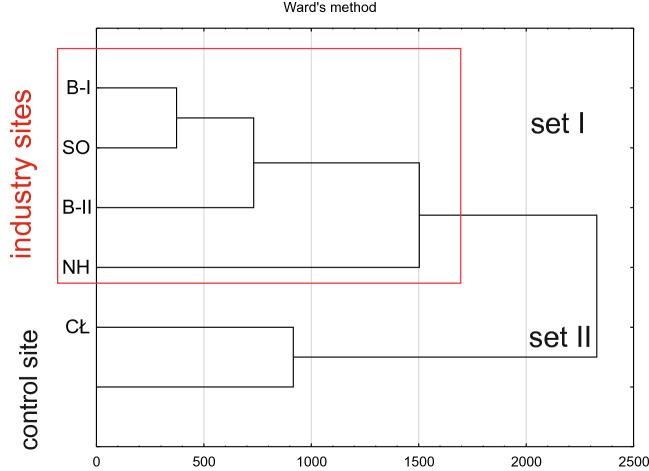
Cluster analysis carried out for the content of all macro- and microelements in lettuce.

The cluster analysis carried out for the content of all macro- and microelements studied showed a clear similarity between lettuce grown in the industrial soils (B-I, B-II, SO and NH), characterized by a higher degree of soil contamination than the samples from the control site and Cło (CŁ) (Fig. 2).

### The degree of metal accumulation in unfertilized lettuce leaves

**Figure 3:**
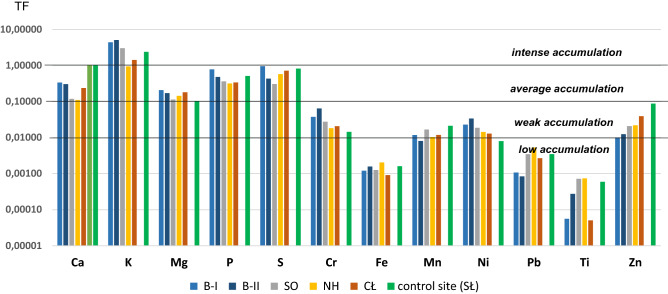
Transfer factor of macro- (Ca, K, Mg, P and S) and microelements (Cr, Fe, Mn, Ni, Pb, Ti and Zn) in lettuce growing on soils from industry areas (B-I, B-II, SO, NH and CŁ) and control site (SŁ).

The highest *TF* values calculated for plants grown in unfertilized soils (variant *a*) were recorded for macroelements (Fig. 3). This is due to the high demand of plants for the main nutrients necessary for their growth and tissue formation. Thus, for all the macroelements analysed (Ca, K, Mg, P and S) the *TF* values exceeded 0.1, both for plants grown in the soils taken from the industrial areas and for those grown in the control soils. This indicates very intense (in the case of K) or moderate (in the case of other macroelements) bioaccumulation. Poor accumulation (0.01 ≤ *TF* < 0.1) was found for such microelements as Cr, Mn, Ni and Zn, whose mean *TF* values decrease in the following order: TF_K_ > TF_P_ > TF_Ca_ > TF_Mg_. Very low bioaccumulation factor (TF ≤ 0.01) was observed for Pb, Fe and Ti.

**Figure 4:**
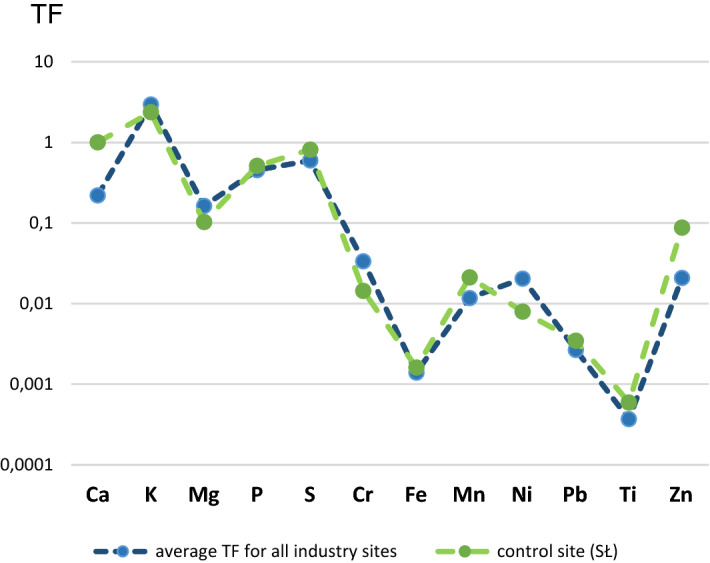
TF average values for industry areas and control site.

When comparing *TF* calculations obtained for the industrial areas with the values calculated for the control site, remarkably similar trends can be noted (Fig. 4). A small difference was found only in the case of K, Mg, Cr and Ni (greater bioaccumulation) and in the case of Ca, P, S, Mn, Zn and Ti (slightly lower bioaccumulation).

The basic macronutrient found in lettuce is K, hence its high bioaccumulation^36^. High *TF* values were also observed for S, which is the basic component involved in photosynthesis and protein synthesis. Together with Mg, it controls N metabolism in the plant and reduces the accumulation of nitrates and nitrites by converting mineral nitrogen into protein. This process is extremely important due to the fact that these compounds can be harmful to humans in excessive amounts^36,37^.

The difference in bioaccumulation of trace elements (i.e. Zn, Pb, Ni or Cr) in the aerial parts of plants grown in soils with varying degrees of contamination may result from the lack of a biological barrier leading to rapid adaptation, or may be the plant’s immune strategy involving the exclusion of these metals (retention in root tissues). There are basically two detoxification mechanisms in the roots. The first involves the deposition of metals in cell walls, and the other—binding metals with phenolic compounds in the vacuoles of tannin cells. The exclusion of excessive amounts of metals is also influenced by the presence of bacteria in the soil, which by increasing the secretion of pectins enhance the sorption capacity, and exclude metals (such as Pb) from the metabolism by binding them in endodermal cells.

### Correlation between the content of elements in unfertilized lettuce leaves

The correlation coefficients (*r*) between the elements contained in lettuce grown in unfertilized soils from the industrial sites showed statistically significant (*p* < 0.05) relationships between: K–Fe, P–Mn, Zn–S, Zn–Cr and Fe–Ti. In addition, the following was found:

- strong correlation (0.5 ≤ *r* < *0.7*) for: Pb–Ca, Pb–Cr, S–Ni, S–Pb, and equally high correlation, but with the opposite sign for: Mn–Pb and S–Ti.
- very strong correlation (0.7 ≤ *r* < 0.9) was observed for: S–Cr, Ca–Ni, Ca–S, Ca–Zn, K–Mg, Ni–Pb, Ni–Zn, P–Mn and Pb–Zn. Similarly strong but inverse correlation was found for: K–Mg, K–Ti and P–Pb.
- nearly complete correlation (0.9 ≤ *r* < 1.0) was found for: Cr–Ca, Cr–Ni, Cr–Zn, Fe–Ti and S–Zn. A nearly complete but inverse correlation was recorded in the case of Fe–K (Table 2).

**Table 2:** Correlation coefficients (r_x,y_) between the elements’ contents in lettuces growing on unfertilized soil from industry area.

	Ca	K	Mg	P	S	Cr	Fe	Mn	Ni	Pb	Ti	Zn
Ca	1.00	− 0.35	0.35	− 0.28	**0.81**	***0.92***	0.16	0.10	**0.86**	*0.59*	0.07	**0.86**
K		1.00	− **0.74**	0.26	0.15	− 0.08	− 0.90*	0.21	− 0.27	0.04	− **0.85**	0.13
Mg			1.00	0.21	0.28	− 0.02	0.33	0.47	− 0.11	− 0.32	0.12	− 0.37
P				1.00	− 0.17	− 0.29	− 0.06	0.89*	− 0.46	− **0.84**	− 0.24	− 0.37
S					1.00	**0.77**	− 0.42	0.29	*0.56*	*0.56*	− *0.52*	0.94*
Cr						1.00	0.02	0.02	***0.95***	*0.69*	− 0.02	0.90*
Fe							1.00	− 0.19	0.24	− 0.20	0.97*	− 0.33
Mn								1.00	− 0.24	− *0.60*	− 0.42	0.03
Ni									1.00	**0.75**	0.26	**0.77**
Pb										1.00	− 0.07	**0.78**
Ti											1.00	− 0.36
Zn												1.00

0.5 ≤ r < 0.7 high correlation (italics); 0.7 ≤ r < 0.9 very high correlation (bolded); 0.9 ≤ r < 1.0 almost full correlation (bold italics); *p < 0.05 statistically significant (emphasis).

These results can be interpreted in two ways. Firstly, they indicate similar or different origin (source) of the elements. Secondly, they illustrate mutual relations (antagonistic or synergistic) that occur between individual elements.

As regards the first indicator, adequate for unfertilized soils, the same origin of the elements found in the soils stems from the chemical composition of processed ores in mining and metallurgical plants in Bukowno, Sosnowiec and Nowa Huta. In Bukowno, lead–zinc mineralization is associated with epigenetic ore-bearing dolomites. The elements accompanying Pb–Zn–Fe sulphides include: Ag, As, Cu, Cd, S, Sb, Se, Te and Tl. In turn, the fact that dolomite (CaMg(CO_3_)_2_) and calcite (CaCO_3_) constitute almost 70% of the mineral composition in Polish Zn–Pb deposits^38^ explains the strong correlation between Pb–Zn–Fe and Ca, Mg or S, found in galena (PbS), wurtzite ((Zn,Fe)S) or greenockite (CdS). In terms of minerals, sulphides constitute almost 11% of deposits mined in Bukowno.

The other indicator—the presence of elements in plants and their interrelationships, results from a complex process influenced by e.g. cation exchange through the cell membrane, intracellular transport, and processes taking place in the rhizosphere (production of sugars, amino acids and organic acids by the roots) accompanied by specific microbial activity.

### The content of macro- and micronutrients in the fresh mass of fertilized lettuce

**Figure 5:**
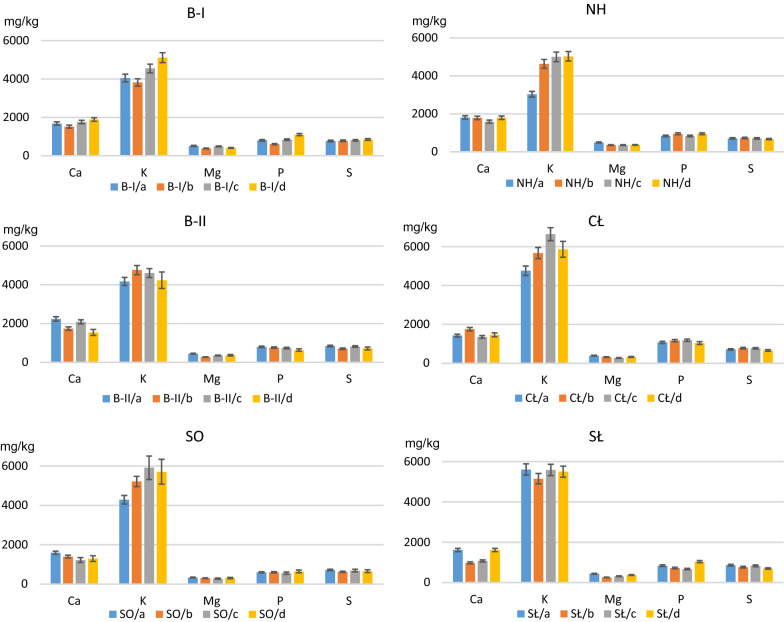
The content of macroelements in lettuce, growing on: *a*—unfertilized soil, *b*—mix of straight fertilizers, *c*—multinutrient fertilizer and *d*—organic fertilizer. B-I, B-II—Bukowno region, SO—Sosnowiec, NH, CŁ—Nowa Huta region, SŁ—control site.

The addition of various types of fertilizers (straight, multinutrient or organic) to the soils collected in the industrial areas caused a different and inconclusive reaction of plants regarding the absorption and accumulation of macroelements in their aerial parts (Fig. 5).

Comparing the content of Ca, K, Mg, P and S in lettuce grown in unfertilized soils from the Zn-Pb ore mining and processing region (Bukowno: B-I and B-II samples) to the soils with the addition of fertilizers: straight (variant *b*), multinutrient (variant *c*) and organic (variant *d*), it was found that:

- the addition of a mix of straight fertilizers caused: a decrease in the content of Ca, P and Mg, and an inconclusive result as regards the K and S content (i.e. a decrease in some of the samples and an increase in others);
- the addition of multinutrient and organic fertilizers caused: an increase in the K content, a decrease in the Mg content and an inconclusive result as regards Ca, P and S (Fig. 5).

Comparing plants grown in the soils from Sosnowiec (SO, the region of coal mining and steel production) and fertilized with variants *b*–*d* to lettuce grown in unfertilized soils (SO, variant *a*), the following was found:

- a lower Ca, Mg and S content, and a higher K content in all three fertilization variants;
- a lower P content after the addition of multinutrient fertilizer and a higher P content after the addition of organic fertilizer.

Samples of lettuce grown in the soils from Nowa Huta (impact of the Fe ore processing industry, samples marked with symbols NH and CŁ) and subjected to the fertilization experiment demonstrated the following:

- a higher K content and a lower Mg content in all three fertilization variants;
- an increase in the Ca, P and S content after the addition of a mix of straight fertilizers;
- a decrease in the Ca content after the addition of multinutrient fertilizer;
- a decrease in the S content after the addition of organic fertilizer and an inconclusive result for P and Ca.

In lettuce grown in the control soils (SŁ, without the influence of any industry and used for agriculture), the following was observed:

- a decreased Ca, K, Mg and S content after the addition of all three types of fertilizers;
- a decreased P content after the addition of straight and multinutrient fertilizers, and an increased P content after the addition of organic fertilizer.

In plants grown in the control soils, the mean content of Ca, K, Mg, P and S for all three fertilization variants was: 1220, 5412, 313, 809 and 762, respectively (in mg kg^−1^ fresh mass). Comparing these results with the data for unfertilized control soils presented in Table 1 (1614, 5610, 438, 839 and 857, respectively; in mg kg^−1^ fresh mass), it was found that the content of all macroelements analysed in lettuce decreased as a result of fertilization.

As for lettuce grown in the soils from the industrial areas, the mean content of Ca, K, Mg, P and S for all fertilization variants was 1614, 5117, 344, 840, 729, respectively (in mg kg^−1^ fresh mass), which in comparison with the mean value calculated for plants grown in the same soils but without fertilization (Table 1) permits to conclude that the addition of fertilizers to the soils from the industrial areas caused an increase in the K and P content and a decrease in the Ca, Mg and S content.

For lettuce grown in the control and fertilized soils, a decrease in the content of all macroelements was found as compared to unfertilized lettuce.

The microelements analysed with respect to their content, depending on the fertilization method used, were divided into two groups based on the range of occurrence of these elements in plants. The first group included: Fe, Mn and Zn. These elements are components of numerous enzymes, they are necessary for plant development and participate in the processes of energy transformation needed for photosynthesis (Fe, Mn), stimulate the formation of chlorophyll (Fe), play a role in the metabolism of carbohydrates (Fe), proteins, phosphorus compounds (Zn, Fe), participate in the reduction of nitrates (Fe), regulate the proportions of ingredients at the cell level (Fe, Zn) or regulate redox reactions (Mn, Fe).

**Figure 6:**
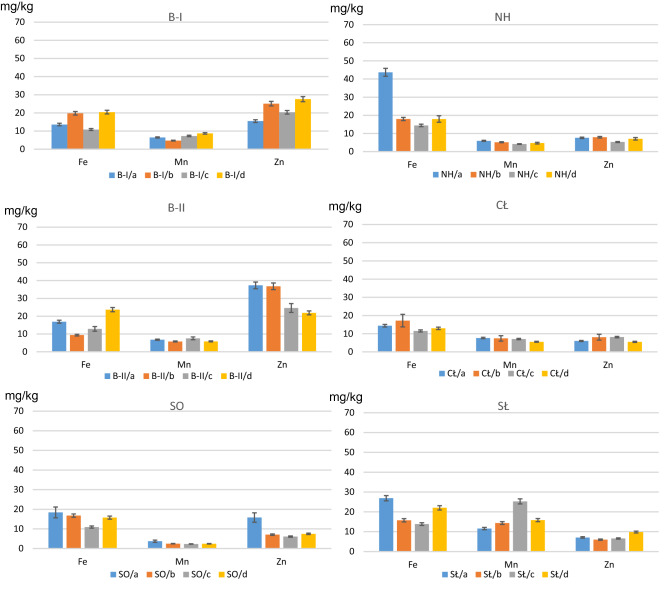
The content of Fe, Mn and Zn in lettuce, growing on: *a*—unfertilized soil, *b*—mix of straight fertilizers, *c*—multinutrient fertilizer and *d*—organic fertilizer. B-I, B-II—Bukowno region, SO—Sosnowiec, NH, CŁ—Nowa Huta region, SŁ—control site.

In lettuce grown in the soils from Bukowno (B-I, B-II) and subjected to fertilization, there were changes in the content of Fe, Mn and Zn as compared to lettuce grown in the same soils, but not fertilized:

- inconclusive results with regard to the content of all three metals after the addition of straight and multinutrient fertilizers (Fig. 6),
- an increase in the Fe, Mn and Zn content in B-I sample, and a decrease in the Fe, Mn and Zn content in B-II sample after the addition of organic fertilizer.

The following was observed for plants grown in the soils from Sosnowiec (SO) and Nowa Huta (NH, CŁ):

- a significant decrease in the Fe, Mn and Zn content in all three fertilization variants as compared to unfertilized samples.

For lettuce grown in the control soils (SL), a decrease in the Fe content and an increase in the Mn content were noted in all three fertilization variants. Also, there was a decrease in the Zn content after the addition of straight and multinutrient fertilizers and an increase in the Zn content after organic fertilization (Fig. 6).

Comparing the mean content of Fe, Mn and Zn in lettuce grown in unfertilized soils with the mean calculated for all soils from the industrial areas and fertilized with all three types of fertilizers, it was found that the content of these elements decreased as a result of adding fertilizers to the soils. None of the plants tested exceeded the so-called normal Fe, Mn and Zn content of 130, 29 and 44–73 mg kg^−1^ DM, respectively^32^.

**Figure 7:**
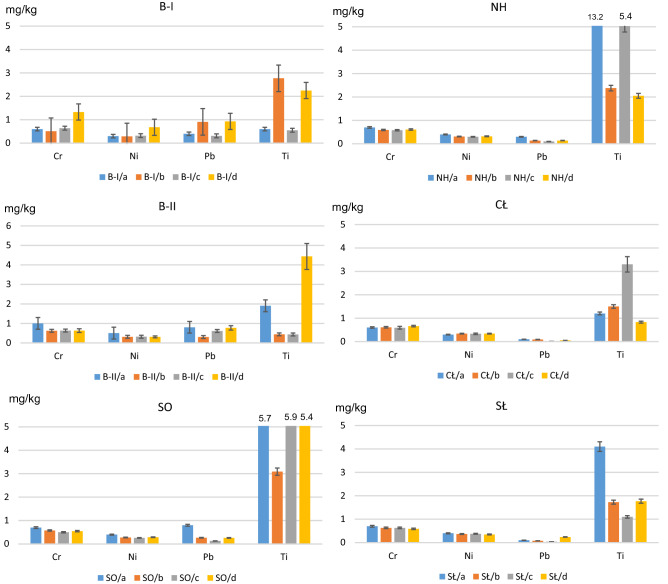
The content of Cr, Ni, Pb and Ti in lettuce, growing on: *a*—unfertilized soil, *b*—mix of straight fertilizers, *c*—multinutrient fertilizer and *d*—organic fertilizer. B-I, B-II—Bukowno region, SO—Sosnowiec, NH, CŁ—Nowa Huta region, SŁ—control site.

The other group of micronutrients included: Cr, Ni, Pb, Ti (Fig. 7). Their content in plants is definitely lower, usually at the level of a few to several ppm, and the necessity of their participation in the normal plant growth has not yet been clearly confirmed. The uptake of these elements is passive and usually proportional to the occurrence of their soluble forms in the soil. However, their excess can be toxic to plants as manifested by chlorotic and necrotic spots.

In the case of lettuce grown in the soils collected from the area of Zn-Pb ore extraction and processing (samples B-I and B-II), the addition of straight, multinutrient and organic fertilizers affected the content of Cr, Ni, Pb and Ti (Fig. 7).

As for lettuce grown in the soils from the area of hard coal mining (sample SO), a noticeable decrease in the content of all microelements was observed, as was the case with the NH sample.

Similar content of Cr, Ni and Pb was found for the last area analysed (CŁ), whereas in the case of Ti the measurement results varied. The addition of straight and multinutrient fertilizers increased the Ti content in lettuce, while the addition of organic fertilizer caused a decrease in the content of this metal by approx. 30% (Fig. 7).

Lettuce grown in the control soils (SŁ) and subjected to fertilization, demonstrated lower Ti, Cr, Ni, Pb content in all three fertilization variants as compared to plants grown in the same soils, but without fertilization.

Comparing the mean content of Cr, Ni, Pb, Ti in lettuce grown in the soil samples taken from the industrial areas and subjected to fertilization (all three fertilization variants) to the results obtained for unfertilized soils, it was observed that the addition of fertilizers caused a decrease in the content of Cr, Ni, Pb, Ti in aerial lettuce leaves.

**Figure 8:**
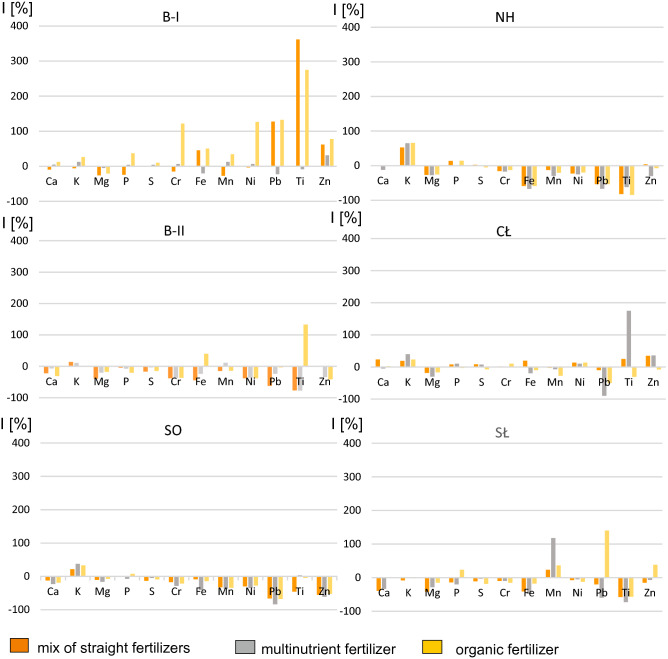
Inhibition rates (I) of macro- and microelements content in lettuce after applying various types of fertilization. B-I, B-II—Bukowno region, SO—Sosnowiec, NH, CŁ—Nowa Huta region, SŁ—control site.

To conclude, the addition of various types of fertilizers to the soils from the industrial areas increased the content of K and P in lettuce. These are the dominant components of fertilizers, which are easily available to plants. Thus, their sorption resulted in a more effective binding of microelements (metals) with the mineral components of the soil and its organic matter, which ultimately led to a decrease in the amount of phytoavailable forms of metals (i.e. Cr, Ni, Pb, Ti) and their amount in the aerial parts of lettuce (Fig. 8).

### Correlation between the content of elements in unfertilized lettuce leaves

As regards the soils collected in the industrial areas and subjected to fertilization with a mix of straight fertilizers, three pairs of elements: Cr–Pb, Mn–Ni and Ni–P, turned out to be statistically significant (at *p* < 0.05). A strong correlation (0.5 ≤ *r* < 0.7) occurred in the case of Ca–Cr, Cr–P, K–Ni, K–P, Mg–Pb, Mg–S and Mg–Ti. Similarly strong but inverse correlation was found for: Pb–Ca, Cr–Fe, Cr–Ti, Fe–Zn, K–Mg, K–Zn, Mn–Ti, Ni–Pb, P–Pb and Ti–Zn. A very strong correlation (0.7 ≤ *r* < 0.9) was observed for the following pairs of metals: Ca–Mn, Ca–Ni, Ca–P, Cr–K, Cr–Ni, Fe–Mg, Fe–Ti, Mn–P, Mn–S and Ni–P. Similarly strong inverse correlation was found for the following pairs of elements: Ca–Ti, Cr–Mg, Cr–Pb, K–Pb and Ni–Ti. A nearly complete correlation (0.9 ≤ *r* < 1.0) occurred for one pair of elements: Mn and Ni (Table 3a).

**Table 3:** Correlation coefficients (r_x,y_) between the elements’ contents in lettuces samples growing on soil from industry areas and fertilized by (A) mix of straight fertilizers. (B) multinutrient fertilizer. (C) organic fertilizer.

Element	Ca	Cr	Fe	K	Mg	Mn	Ni	P	Pb	S	Ti	Zn
**A**												
Ca	1.00											
Cr	*0.52*	1.00										
Fe	− 0.33	− *0.69*	1.00									
K	0.19	**0.80**	− 0.22	1.00								
Mg	− 0.06	− **0.80**	**0.86**	− *0.60*	1.00							
Mn	**0.82**	0.35	− 0.18	0.26	− 0.01	1.00						
Ni	**0.80**	**0.71**	− 0.36	*0.63*	− 0.35	0.90*	1.00					
P	**0.79**	*0.54*	0.03	*0.59*	− 0.01	**0.82**	0.89*	1.00				
Pb	− *0.51*	− 0.89*	0.32	− **0.84**	*0.51*	− 0.27	− *0.64*	− *0.68*	1.00			
S	0.47	− 0.36	0.36	− 0.30	*0.59*	**0.75**	0.39	0.47	0.33	1.00		
Ti	− **0.70**	− *0.66*	**0.83**	− 0.25	*0.61*	− *0.69*	− **0.74**	− 0.41	0.34	− 0.19	1.00	
Zn	0.11	− 0.07	− *0.64*	− *0.51*	− 0.27	0.16	− 0.03	− 0.39	0.48	0.15	− *0.60*	1.00
**B**												
Ca	1.00											
Cr	**0.83**	1.00										
Fe	0.35	0.20	1.00									
K	− **0.82**	− *0.61*	− 0.31	1.00								
Mg	*0.58*	**0.70**	− 0.07	− **0.81**	1.00							
Mn	*0.66*	0.89*	− 0.08	− 0.26	0.42	1.00						
Ni	*0.56*	0.88*	0.08	− 0.17	0.36	0.96*	1.00					
P	− 0.09	0.40	0.03	0.44	− 0.09	*0.59*	**0.76**	1.00				
Pb	0.90*	*0.57*	0.09	− **0.75**	0.44	0.48	0.29	− 0.39	1.00			
S	**0.75**	0.88*	− 0.20	− 0.42	*0.54*	0.96*	**0.85**	0.35	*0.66*	1.00		
Ti	− **0.83**	− 0.89*	0.15	*0.57*	− *0.64*	− 0.90*	− **0.77**	− 0.20	− **0.75**	− 0.98*	1.00	
Zn	**0.88**	*0.57*	0.14	− *0.56*	0.23	*0.58*	0.41	− 0.20	0.95*	**0.70**	− **0.75**	1.00
**C**												
Ca	1.00											
Cr	**0.71**	1.00										
Fe	0.44	0.31	1.00									
K	− 0.39	− 0.09	− 0.95*	1.00								
Mg	0.93*	**0.79**	*0.68*	− *0.59*	1.00							
Mn	**0.76**	**0.87**	0.44	− 0.34	**0.86**	1.00						
Ni	**0.70**	0.99*	0.26	− 0.03	**0.77**	**0.84**	1.00					
P	*0.66*	*0.63*	− 0.31	0.34	0.47	*0.64*	*0.65*	1.00				
Pb	0.46	**0.71**	**0.83**	− *0.64*	**0.74**	*0.69*	*0.67*	− 0.04	1.00			
S	**0.71**	0.96*	*0.55*	− 0.33	**0.87**	**0.88**	0.95*	0.44	**0.87**	1.00		
Ti	− *0.50*	− 0.31	0.38	− 0.28	− 0.30	− *0.51*	− 0.32	− 0.90*	0.26	− 0.14	1.00	
Zn	*0.54*	**0.77**	**0.81**	− *0.63*	**0.80**	**0.78**	**0.72**	0.08	0.99*	0.90*	0.13	1.00

Designations as in the Table 2.

The following pairs of metals proved to be statistically significant (at *p* < 0.05) for multinutrient fertilization: Ca–Pb, Cr–Mn, Cr–Ni, Cr–Ti, Mn–Ni, Mn–S, Mn–Ti, Pb–Zn and S–Ti. A strong correlation (0.5 ≤ *r* < 0.7) was obtained for: Ca–Mg, Ca–Mn, Ca–Ni, Cr–Pb, Cr–Zn, K–Ti, Mg–S, Mn–P, Mn–Zn and Pb–S. A similarly strong but inverse correlation was found for the following pairs of elements: Cr–K, K–Zn and Mg–Ti. A very strong correlation (0.7 ≤ *r* < 0.9) occurred in the case of Ca–Cr, Ca–S, Ca–Zn, Cr–Mg, Cr–Mn, Cr–Ni, Cr–S, Ni–P, Ni–S and Zn–S. An equally strong but inverse correlation was observed for: Ca–K, Ca–Ti, Cr–Ti, K–Mg, K–Pb, Ni–Ti, Pb–Ti and Ti–Zn. A nearly complete correlation (0.9 ≤ *r* < 1.0) was found in the case of Ca–Pb, Mn–Ni, Mn–S and Pb–Zn. A strong inverse correlation was observed for two pairs of metals i.e. Mn–Ti and S–Ti (Table 3b).

In the case of organic fertilizer, the following pairs of elements proved to be statistically significant (at *p* < 0.05): Ca–Mg, Cr–Ni, Cr–S, Fe–K, Ni–S, P–Ti, Pb–Zn and S–Zn. A strong correlation (0.5 ≤ *r* < 0.7) was found for Ca–P, Ca–Zn, Cr–P, Fe–Mg, Fe–S, Mn–P, Mn–Pb, Ni–P and Ni–Pb. A similarly strong inverse correlation was found for: Ca–Ti, K–Mg, K–Pb, K–Zn and Mn–Ti. A very strong correlation (0.7 ≤ *r* < 0.9) was observed for the following pairs of metals: Ca–Cr, Ca–Mn, Ca–Ni, Ca–S, Cr–Mg, Cr–Mn, Cr–Pb, Cr–Zn, Fe–Pb, Fe–Zn, Mg–Mn, Mg–Ni, Mg–Pb, Mg–S, Mg–Zn, Mn–Ni, Mn–S, Mn–Zn, Ni–Zn and Pb–S. A nearly complete correlation (0.9 ≤ *r* < 1.0) was found for the following pairs of elements: Ca–Mg, Cr–Ni, Cr–S, Ni–S, Pb–Zn and S–Zn. A nearly complete inverse correlation was found for Fe–K and P–Ti (Table 3c).

The most important factors affecting the absorption of elements by plants include: the total content of elements available in the soil, their concentration in the soil solution and mutual quantitative proportions. Antagonistic and synergistic reactions occur between the main elements (Ca, Mg, P, K, S, N, Cl, Na and Si) and trace elements absorbed by plants. And so, for Ca, synergism with Cu, Mn or Zn is noted. The Ca–Zn dependence is extremely important from the point of view of the absorption of Zn from the soil solution by the plant. In turn, for Mg there is a noticeable synergism with Al and Zn, and antagonism with Cr, Mn, Zn, Ni or Fe. For P, synergism with Al, Cu, F, Mn or Zn is observed, and antagonism with As, Cd, Cr, Fe, Mn, Ni, Pb, Zn. Physiologically important antagonism exists between Fe and P, since an excess of Fe causes a deficiency of P and K^32^. In turn, Pb uptake and activity in plants are antagonistically influenced by the presence of Ca, S and P. These elements cause precipitation of Pb in poorly soluble forms both in the root environment and aerial tissues. On the other hand, Pb–Zn interdependence consists in mutual transport disturbances from the roots to the aerial parts of plants. Titanium, on the other hand, is particularly concentrated in the Fe–Mn concretions and belongs to the elements with the lowest phytoaccumulation index, which has been demonstrated in Table 3.

### Fertilizers and metal content in lettuce leaves

The content of elements in lettuce is undoubtedly determined by the method of soil fertilization. This has been shown by comparing the amount of metals between fertilized (variants *b*-*d*) and unfertilized lettuce.

In lettuce grown in the soil from Bukowno (site B-I), a decrease in the amount of Ca, Mg, P, Cr, Mn and Ni was noted after the addition of a mix of straight fertilizers (by − 10%, − 26%, − 24%, − 15%, − 27% and − 3%, respectively). However, there was an increase in the S, Fe, Pb, Ti and Zn content (Fig. 8). In the case of multinutrient fertilization, the amount of Fe, Mg, Pb and Ti decreased by − 20%, − 5%, − 29% and − 8%, respectively. As for organic fertilization, there was no decrease in metal content as compared to the unfertilized soil, with the exception of Mg (− 20%) which is one of the elements conducive to lettuce growth. In lettuce grown in the fertilized soil from site B-II in Bukowno, the content of elements decreased significantly as compared to the unfertilized soil. This trend was observed for almost all elements and fertilization variants (Fig. 8). An increase in content was noted only for K (all three fertilization variants), Mn (multinutrient fertilization) and Ti (organic fertilization). The largest decrease (up to − 78%) was recorded for Ti in lettuce fertilized with multinutrient fertilizer and a mix of straight fertilizers. A large decrease (about 66%) was also found for Pb in lettuce fertilized with a mix of straight fertilizers. A very similar significant decrease was also observed in the case of Cr and Ni. In all fertilization variants their content fluctuated in the range of 36–38% (Fig. 8).

Almost the same situation as in the case of site B-II was observed in lettuce grown in the soils from Sosnowiec. There was a decrease in content for almost all elements and fertilization variants, except for 22–38% increase in the content of K (all fertilization variants), 0.5% and 8% increase in P in lettuce fertilized with a mix of straight fertilizers and manure, respectively, and a slight increase of 3.3% in Ti in lettuce fertilized with multinutrient fertilizer. The elements whose content increased are useful for plant growth, as they build plant tissues and play a yield-enhancing role. The largest decrease was recorded in the following metals after multinutrient fertilization: Pb (− 83%), Zn (− 61%), Ni (− 30%), Cr (− 32%), Fe (− 41%) and Mn (− 38%).

In lettuce grown in the soils from Nowa Huta, as in the previous case, a decrease in content was observed for most elements and fertilization variants (Fig. 8). The exception were two macroelements K and P, whose contents increased by 61% and 9%, respectively. In the case of straight fertilizer, the S and Zn content increased by several percent. The largest decrease, by 82% and 84%, was observed for Ti (for multinutrient and organic fertilizer, respectively). A slightly smaller decrease was found for Fe (59–67%) and Pb (55–66%) in all three fertilization variants, with the highest reduction recorded for multinutrient fertilizer. With this type of fertilization, there was also a decrease in the content of Zn, Mn, Cr and Ni by: − 30%, − 30%, − 20% and − 24%, respectively.

As regards lettuce grown in the soils collected in Cło, there was a decrease in the content of Mg, Fe, Mn and Pb (all three fertilization variants), on average by − 22%, − 3%, − 12% and − 50%, respectively. An increase in accumulation was observed after fertilization with:

- a mix of straight fertilizers for: Ca, K, P, S, Cr, Fe, Ni, Ti and Zn by: 23%, 19%, 8%, 8%, 2%, 19%, 13%, 25% and 35%, respectively,
- multinutrient fertilizer for: K, P, S, Ni, Ti and Zn by: 39%, 10%, 8%, 10%, 175% and 36%, respectively,
- organic fertilizer for: Ca, K, Cr and Ni by: 2%, 23%, 10% and 13% respectively.

In lettuce grown in the control soils from Słopnice, a decrease in the content of most of the macro- and microelements analysed was observed for all three fertilization variants (Fig. 8). An increase was recorded only for Mn, whose content in lettuce increased in all three types of fertilization (from 23 to 118%). Also, the content of P, Pb and Zn increased, by 23%, 140% and 38%, respectively, after the addition of organic fertilizer. A significant reduction in the content of elements in lettuce leaves was observed after multinutrient fertilization, especially for Ti and Pb (by − 73% and − 56%, respectively). A slight decrease was also found for Ni (14–21%) and Cr (13–18%) in all fertilization variants.

## Discussion

### The content of macroelements and microelements in lettuce samples – the comparison with FoodData Central

The comparison of the study results with the US Department of Agriculture data revealed a significant excess of the maximum content published by this agency in the material analysed (Table 4). This applies especially to Ca, Mg, Zn, P and K^36^. Only in some lettuce samples, the Fe and Mn content fell within the recommended range, although in the case of Fe, the maximum value was exceeded in samples: B-I/*a*, B-I/*c*, B-II/*c*, NH/*d*, SŁ/*c* and SŁ/*d*, and in the case of Mn in samples: SO/*a*, SO/*b* and SO/*c*. Reference values were not provided for Cr, Ni, Pb and Ti.

**Table 4:** The content of microelements in lettuce leaves—a literature review.

Authors	Type	Parameter	Cr	Fe	Mn	Ni	Pb	Zn
			[mg kg^−1^ DM]					
This study	Polluted samples	Range	6.9–12.1	160.3–513.7	43.9–89.2	3.5–6.0	1.2–10.2	70.5–439.2
	Control sample		8.5	316.4	136.0	5.2	1.2	83.0
Kabata-Pendias and Pendias^32^	Physiological content for plants	Range	0.1–0.5	–	30–300	0.1–5	5–10	25–150
	Toxic content for plants	Range	5–20	–	400–1000	10–100	30–300	100–400
	Leafy wegetables	Range	5–15	–	10–130	0.5–5	2–20	80–120
	Lettuce leaves	Av./range	0.8	–	29	1–3.8	0.7–3.6	44–77
U.S. Department of Agriculture^1^		Av	–	101.2	29.4	–	–	21.2
		Range	–	36.5–222.3	16.5–38.8	–	–	14.1–27.1
Pančevski et al.^41^	Lettuce leaves (n= 3)	Range	0.29–0.55	41–159	8.03–41.0	0.67–1.99	2.1–5.6	21.8–37.5
	Transfer factor*	Range	0.1	0.002–0.017	0.02–0.08	0.02–0.05	0.01–0.04	0.09–0.01
Medunić et al.^42^	Lettuce leaves	Av	5.48	–	–	–	2.84	30.2
	Transfer factor	Av	0.03	–	–	–	0.02	0.03
Semical et al.^43^—unfertilized lettuce	Growed at polluted soil	Av	–	–	–	–	50.1	422.4
	Transfer Factor	Av	–	–	–	–	0.01	0.4
	Growed at control soil	Av	–	–	–	–	17.9	416.0
	Transfer factor	Av	–	–	–	–	0.03	2.34
Semical et al.^43^—fertilized lettuce	Growed at polluted soil	Av	–	–	–	–	107.8	8.36
	Control growed at control soil	Av	–	–	–	–	0	19.8

The study results were also compared to the physiological and toxic content proposed by Kabata-Pendias and Pendias^32^ (Table 4). In all lettuce samples analysed, the Cr concentration was at a toxic level, while the amount of Mn was at the physiological level. Ni (B-II/*a*, SŁ/*a*, B-I/*d*) and Pb (B-I/*b*,*d*, B-II/*a*) were present in elevated amounts. The highest Zn content in lettuce grown in the soils from Bukowno and Sosnowiec (samples: B-I/*a,b,c,d*, B-II/*a,b,c, d*, SO/*a*) was also at a toxic level. The Ti content in lettuce leaves found in the samples from Sosnowiec (SO/*a,c,d*, NH/*a,b* and B-II/*d*) also exceeded the natural content.

The fact that there are many different limit values in the literature makes it difficult to conclusively assess metal contamination of lettuce (Table 1). Comparing the obtained content of Pb in unfertilized lettuce to the permissible limit provided by Tasrina et al.^39^ and Latif et al.^33^, 0.3 mg kg^−1^, or by Islam et al.^31^, 0.1 mg kg^−1^, or by Ramteke et al.^40^, 0.2 mg kg^−1^, it was found that it was exceeded in nearly of the cases for all the samples examined. The only exception was lettuce grown in the soil from Cło (CŁ), Nowa Huta (NH) and the control soil (SŁ) for the limit of 0.3 mg kg^−1^. In the case of Zn, the permissible limit of 10 mg kg^−1^ indicated by Kabata-Pendias and Pendias^32^ was exceeded only for lettuce grown in the soils from Bukowno (B-I, B-II) and Sosnowiec (SO). In turn, considerably higher permissible Zn levels provided by Tasrina et al.^39^ − 60 mg kg^−1^, Latif et al.^33^ − 100 mg kg^−1^, and Ramteke et al.^40^ − 100 mg kg^−1^, were not exceeded in any lettuce grown in unfertilized soils. The permissible limits (data in brackets given in mg kg^−1^) for Cr (2.3), Fe (425), Mn (500) and Ni (1.5 or 10) were not exceeded in any of the lettuce samples tested. On the other hand, the levels of Ni (0.1 mg kg^−1^) and Mn (0.2 mg kg^−1^) were exceeded in all samples of unfertilized lettuce.

### Metallurgy industry (Bukowno)

Pollution from the metallurgy industry occurs in many regions around the world, one of them being the city of Veles in Macedonia, where the Zn–Pb smelter “Zletovo” used to operate. Research by Pančevski et al.^41^ shows that the smelter was a significant emitter of pollutants (mainly metals) such as: Cd, Pb, Zn, In, Hg, As, Sb. Comparing the contents of Zn, Pb, Ni, Mn, Cr and Fe in samples of the unfertilized soil from Bukowno and lettuce grown in that soil with the results obtained by Pančevski et al.^41^, it was found that the concentration of Zn, Pb, Mn in Bukowno soils was almost 10 times higher than in the soils from Veles (Table 5). This translates into the metal content in lettuce leaves, because these elements are proportionally absorbed by the root system. This is evidenced by the values of the bioaccumulation factor TF, which are comparable in lettuce grown in the soils from Bukowno and the soils from Veles. In the case of Ni, Cr and Fe, the content of these metals in the soils from Bukowno is lower. However, an inverse correlation is observed in lettuce, which is also reflected in the *TF* values. The difference may be due to the climate characteristics of the Balkan Peninsula and different lengths of the growing season in the areas compared. According to Kabata-Pendias and Pendias^32^, the uptake and transport of Cr by plants is closely related to the Fe content in the plant and is manifested by a more or less constant Cr:Fe ratio. For the samples from Veles and Bukowno, the Cr:Fe ratio is 0.003–0.007 and 0.43–0.61, respectively. According to the abovementioned authors, the Cr content in greenhouse vegetables is almost an order of magnitude higher than the amount found in field vegetables, which would confirm the results obtained for lettuce grown in the soils from Bukowno. In turn, the Ni content in plant tissues is generally inversely proportional to the Fe content. Differences in the uptake of these elements may result from soil properties and synergistic-antagonistic interactions between elements. The soils from Bukowno and Veles also differed in terms of the content of macroelements. In the latter soils, the Ca, Mg and K content was significantly higher than in the soils from Bukowno (except for P).

**Table 5:** The content of macroelements in lettuce leaves—a literature review.

Authors	Type	Parameter	Ca	K	Mg	P
			[mg kg^−1^ DM]			
This study	Polluted samples	Range	16,749.0–26,354.4	35,686.3–55,980.3	3948.1–6057.0	7053.3–12,642.4
	Control sample		18,999.4	66,000.0	5149.4	9874.1
U.S. Department of Agriculture^1^	Lettuce leaves	Av	4235	22 823	1529	3412
		Range	2588–5294	13,647–30,941	706–2000	2000–4235
Pančevski et al. (2014)	Lettuce leaves	Range	3822–14,635	12,844–27,323	3768–4452	1540–2840
	Transfer factor*	Range	0.1–0.9	0.9–1.9	0.4–0.6	1.5–3.1

^1^fresh mass converted by dry mass using the conversion factor of 0.085;

*data calculated;

“– “ lack data.

### Coal industry (Sosnowiec)

Soil pollution associated with coal mining and combustion has been the subject of numerous scientific studies^42^. In Croatia, Istrian type coal rich in organic sulphur (up to 14% of its weight) was mined in the town of Labin (Raša coalfield) until 1999. Comparing soil and lettuce samples taken by Medunić et al.^42^ from arable fields in the vicinity of the mine, slag storage site, machine tool factory and chromium plate factory with samples from Sosnowiec, it was observed that the Pb content in the latter was almost two times higher than in samples collected in Labin. The opposite was found for Zn. The soils around the Labin hard coal mine were also over six times more contaminated with Cr than the soils from Sosnowiec, which may be attributed to the activity of a chromium plate factory operating in that area. Lettuce grown in the soils from Sosnowiec is “richer” in Pb, Zn and Cr than lettuce grown in the soils from Labin. The values of the bioaccumulation index *TF* indicate that lettuce grown in the soil from Sosnowiec absorbed a greater amount of these metals than lettuce from Labin in relation to the initial content in the soil. This may be due to differences in phytoavailability and a twice higher content of organic matter. This is most likely the result of the complexation or chelation of these metals, making them less available to plants.

The content of Zn in lettuce from Sosnowiec was not much higher than the natural values given by Kabata-Pendias and Pendias^32^. The exception was unfertilized lettuce, where the Zn concentration (184 mg kg^−1^) exceeded the limit (130 mg kg^−1^). In the case of Pb, the content of this metal did not fall within the range proposed by the said authors only in unfertilized lettuce, where it was over twice as high as the limit. The Cr level in all lettuce samples substantially exceeded the 0.8 mg kg^−1^ value proposed by Kabata-Pendias and Pendias^32^. The amount of Mn and Ni in lettuce samples generally did not exceed the limit values given by the said authors, except for unfertilized lettuce.

### Steel works (Nowa Huta)

Comparing the Pb and Zn content in lettuce grown in the soils from Nowa Huta with the content of these metals in vegetables analysed by Szwalec and Mudała^25^, it can be concluded that out of all the vegetables studied (carrots, parsley, beetroot and celery), lettuce accumulated the highest amount of metals in its tissues. This would support the assumption that leafy vegetables are more sensitive and accumulate more heavy metals than other vegetables grown in similar conditions.

Zn accumulation in lettuce from Nowa Huta and Cło was slightly higher than the mean content given by Kabata-Pendias and Pendias^32^ for lettuce grown in Poland. The amount of Pb in both locations fell within the range proposed by the said authors, while the content of Cr and Mn was higher for both sites. Ni content in lettuce from Nowa Huta and Cło was close to the values given by these authors.

### Control site (Słopnice)

Lettuce grown in the control soils had a higher content of all metals as compared to the ranges provided by Kabata-Pendias and Pendias^32^. The exception was Pb, which occurred in amounts from the lower limit of the range. In the case of Cr and Mn, these values were almost 10 times higher. As for Zn and Ni, they slightly exceeded the upper limit indicated by the said authors. These results seem to be surprising, given that the area is free of any industrial activity and transportation. The only threat may be low emissions resulting from hard coal burning in home furnaces. Low emissions are associated with the release of dusts and heavy metals such as Hg, Cd, Pb, Mn, Cr into the atmosphere. However, the area is not intensively developed and there are only a few detached houses in the vicinity of the soil collection site, which are more than 100 m away. The results may also stem from inappropriate agricultural practices and acidic soil pH.

### The effect of fertilization on the metal content in lettuce leaves

The content of heavy metals in vegetables is influenced by the type of soil fertilization. Semical et al.^43^ conducted a pot experiment involving the cultivation of lettuce in contaminated and reference soil, which was collected in the village of Lăpuşel, Romania (Table 4). The study showed that lettuce grown in unpolluted and unfertilized soil was characterized by a greater capacity to accumulate Zn and Pb than lettuce grown in unpolluted but fertilized soil. In the case of contaminated soil, a greater accumulation of Pb occurred in fertilized lettuce, while an inverse relationship was found for Zn. In the present study, similar relationships were observed in the lettuce analysed. A smaller accumulation of Pb was found in lettuce fertilized with a mix of straight fertilizers, multinutrient fertilizer and organic fertilizer in relation to the concentrations found in unfertilized lettuce. However, it should be emphasized that the amounts of Pb in the soils used to grow lettuce were at the level of Pb in the reference soils from Lăpuşel, and the content of Zn in most samples of fertilized lettuce was lower than that in unfertilized plants and varied depending on the type of fertilization used. To compare the results obtained in this study with the findings by Semical et al.^43^, one should rely on the initial content of Zn in the soil. Generally, fertilization increased the amount of Zn in lettuce. Zn content in the soils from Cło and Słopnice was similar to the concentrations in the reference soils from Lăpuşel. Generally, however, a higher Zn content was found in fertilized lettuce than in unfertilized plants. The amount of Zn in the soils from Bukowno (site B-I) was at the level of the contaminated soils in Romania, and fertilized lettuce had a higher concentration of Zn than unfertilized lettuce. Zn pollution of the soils from Sosnowiec and Nowa Huta was at an average level compared to the soils from Romania. Soil fertilization caused a decrease in the Zn content in lettuce leaves. B-II soil presented significantly higher concentrations of Zn than the contaminated soil from Romania, and an increase in the Zn content in fertilized lettuce as compared to unfertilized lettuce was recorded only in case of multinutrient fertilization.

## Conclusions

Appropriate agricultural practices, especially adequate fertilization, play a special role in fields located in the vicinity of industrial areas associated with mining and processing of metal ores. Application of fertilizers to contaminated soils may cause mobilization or immobilization of heavy metals, depending on the properties of the soils and the types of fertilizers used.

The type of fertilization is particularly important in the cultivation of edible plants in kitchen gardens. When growing in contaminated soils, these plants may accumulate metals with varying intensity. Leafy and root vegetables are most sensitive, which has been confirmed by the present study.

Heavy metal uptake by *Lactuca sativa* L varies. In the case of Pb, Zn and Fe, the uptake is proportional to the content of these metals in the soil. However, this species may uptake trace elements in large amounts even if their content in the soil is low. It has been demonstrated that Pb, Zn, Cd and Cr are accumulated in excessive amounts in the lettuce leaves.

*Lactuca sativa* L may be considered a good indicator of metal contamination, and it is recommended not to cultivate this species in areas adjacent to industrial plants.

Appropriate agronomic practice, especially adequate dosing of mineral and organic fertilizers, may contribute to the decrease in heavy metal availability to plants. Organic fertilizers seem to be particularly effective as, due to their sorption capacity, they can efficiently bind trace elements in soil.

The present study has demonstrated that mineral fertilizers also play a role in reducing the amount of phytoavailable forms of heavy metals. However, the decisive factor is the level of soil contamination. It is recommended that before applying fertilizers a detailed examination of the soil’s content of macroelements should be performed in order to avoid over-fertilization.
